# Immunological changes related to oral tolerance

**DOI:** 10.1186/2045-7022-1-S1-S46

**Published:** 2011-08-12

**Authors:** Stephan Strobel

**Affiliations:** 1 UCL Institute of Child Health, London, UK

## Background

The default response to protein antigens in the intestine is the induction of systemic and local hyporesponsiveness (OT). There is increasing interest in the role of dietary manipulation and probiotics in the development and prevention of allergic and other diseases. Little is known how the environment and nutritional factors modulate systemic and local immune responses. This presentation addresses immunological mechanisms at the interface of innate and adaptive immunity that determine how the body responds to orally administered proteins and how local microbiota may modify these.

## Recent findings

There is evidence that dendritic cells in the intestinal mucosa play a particular part in OT induction. They take up dietary proteins, which may be sampled in the lumen and migrate to the draining mesenteric lymph node, where they induce regulatory CD4+ T-cell differentiation. An important role of retinoic acid (Vitamin A) in this event has been identified. The regulatory properties of (tolerised) T cells are discussed and it is proposed that the gut microenvironment maintains homeostasis by conditioning dendritic cells to remain in a quiescent state. Inhibitory signalling via Toll-like-Receptors (TLRs) by commensal bacteria possibly contributes to this process.

## Conclusion

A regulatory innate and adaptive network controls how dietary antigens are taken up and presented to T lymphocytes by specialized antigen-presenting cells. Elucidating their nature and how they are influenced by external factors, including changes in the host’s microbiota may help develop novel therapies for allergy and help understand diseases such as coeliac disease. Current immunotherapeutic approaches to specific oral tolerance induction (SOTI) will be discussed within the framework of oral tolerance induction in humans. Recent advances in our understanding of oral tolerance, the interactions between the innate and adaptive immune system, and the way in which intestinal microbiota may affect the outcome of intestinal antigen exposure will be addressed.

